# A Case of Five-Year Survival After Combined-Modality Treatment for Non-Small Cell Lung Cancer With Intraspinal Metastasis

**DOI:** 10.7759/cureus.20960

**Published:** 2022-01-05

**Authors:** Carsten Nieder, Bård Mannsåker

**Affiliations:** 1 Oncology, Nordland Hospital Trust, Bodø, NOR

**Keywords:** 5-year overall survival, long-term outcome, immune check-point inhibitor, cytotoxic chemotherapy, radiotherapy (rt), central nervous system metastasis, intradural extramedullary spinal metastasis, metastatic non-small cell lung cancer

## Abstract

This case report describes the treatment approach and outcome in a 69-year-old female patient with non-small cell lung cancer (NSCLC) diagnosed with T4 N2 M1b (intraspinal) disease. The two most common targets for tyrosine kinase inhibitors (epidermal growth factor receptor and anaplastic lymphoma kinase) were not expressed. Programmed death-ligand 1 (PD-L1) was expressed in <50% of the tumor cells. In 2016, initial guideline-concordant treatment with carboplatin/vinorelbine chemotherapy was initiated. Between the first two cycles, all positron emission tomography (PET) positive lesions were irradiated with 30 Gy in 10 fractions (lung, nodes, thoracic spinal manifestation). After nine months with excellent response (at least partial remission, possibly fibrosis only), bilateral lung metastases were diagnosed. The patient was started on nivolumab monotherapy (later atezolizumab due to a change in National practice) and completed two years of treatment. She is currently in continued complete remission with regular follow-up examinations. This case illustrates that outcomes comparable to those observed in patients with limited brain metastases may be observed in patients with localized intraspinal disease and that immune checkpoint inhibitors play an important role in the management of metastatic NSCLC.

## Introduction

Metastatic non-small cell lung cancer (NSCLC) continues to represent a formidable challenge for the different disciplines involved in cancer care despite significant advantages that have led to improved outcomes [1]. One of the factors responsible for limited long-term survival rates is the development of central nervous system (CNS) metastases, which typically are located in the brain or meninges. Their multidisciplinary treatment involves different radiotherapy modalities, surgical resection, and systemic therapy [2-8]. Defined subtypes of NSCLC, e.g., those with epidermal growth factor receptor (EGFR) mutations or anaplastic lymphoma kinase (ALK) translocations, may respond to targeted agents; however, wild-type NSCLC (negative EGFR, ALK, and other common alterations) is the prevailing tumor type in the authors' geographical region [9]. Thus, chemotherapy and immune checkpoint inhibitors (ICIs) are the mainstay of systemic therapy. Compared to brain metastases, the presence of intraspinal metastases at the time of the initial diagnosis is uncommon [10,11]. Especially, isolated intraspinal seeding detected during upfront radiological work-up represents an unusual starting point. It is therefore informative to report clinical experiences with different types of treatment in the current era of ICIs.

## Case presentation

**Figure 1:**
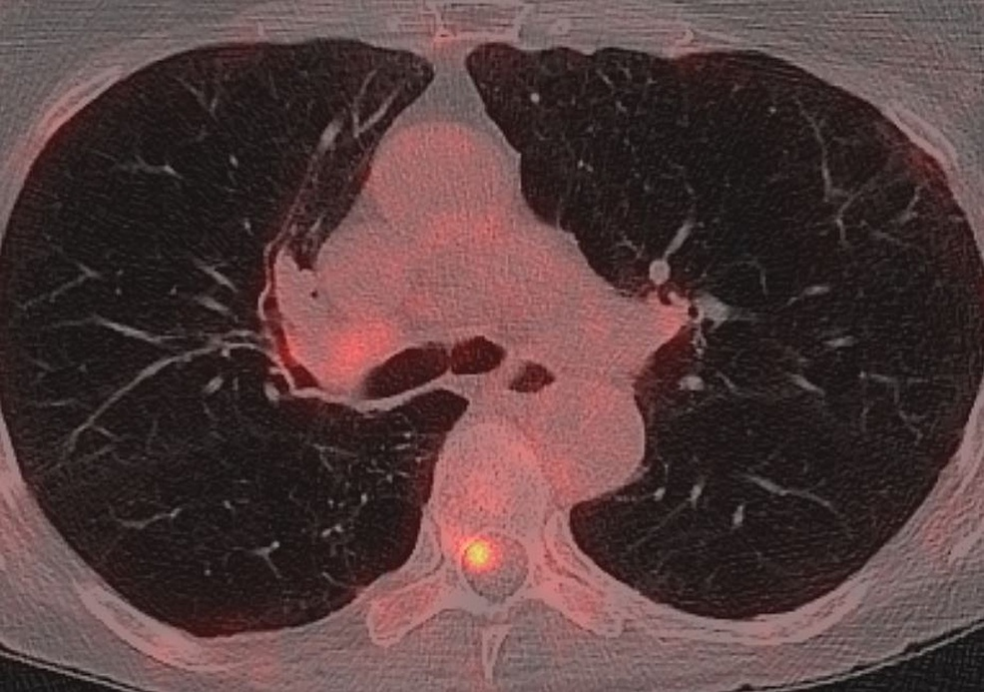
Axial PET-CT scan (tracer 18F-fluorodeoxyglucose) showing intraspinal tracer uptake at the level of the seventh thoracic vertebra, clearly separated from the lung primary. PET-CT, positron emission tomography-computed tomography

**Figure 2:**
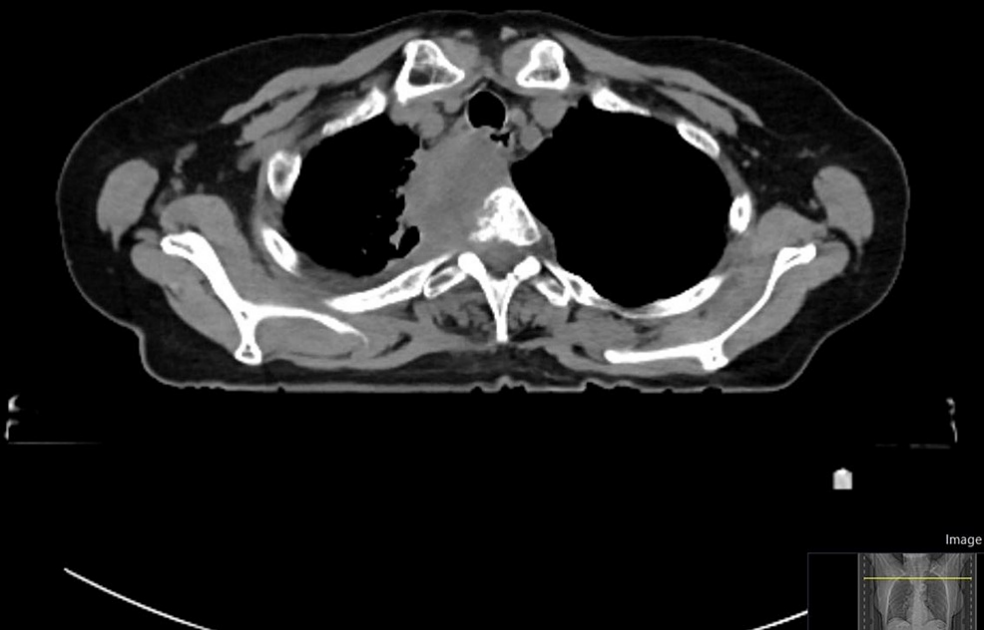
Axial CT scan at the level of the primary lung tumor in the right upper lobe. CT, computed tomography

In August 2016, a 69-year-old female patient presented to her primary care physician with pain in the right shoulder. An X-ray examination led to a suspicion of a lung tumor, resulting in referral to chest computed tomography (CT) and a pulmonologist, who also ordered a positron emission tomography (PET)-CT scan and brain magnetic resonance imaging (MRI). The patient was a former smoker (at least 40 pack-years) with known chronic obstructive pulmonary disease and lung emphysema (no other comorbidity, performance status 1). She did not report any weight loss, and neurological deficits were absent. The blood test results were largely unremarkable, except for elevated C-reactive protein (24 mg/L). Lactate dehydrogenase (LDH) was normal. Figures 1, 2 show the PET-CT and CT findings in the spinal canal and right lung, respectively.

**Figure 3:**
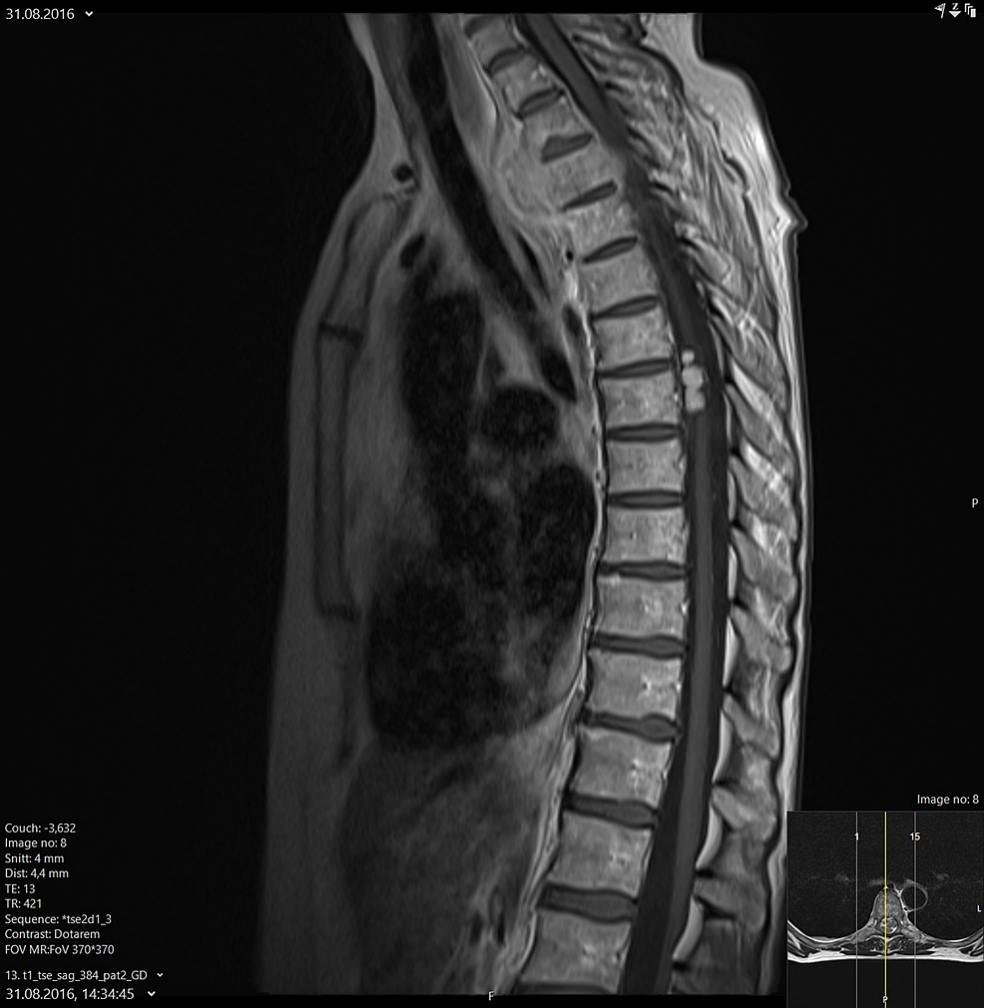
Sagittal contrast-enhanced T1 TSE MRI scan of the intraspinal lesion. MRI, magnetic resonance imaging; TSE, turbo spin echo

**Figure 4:**
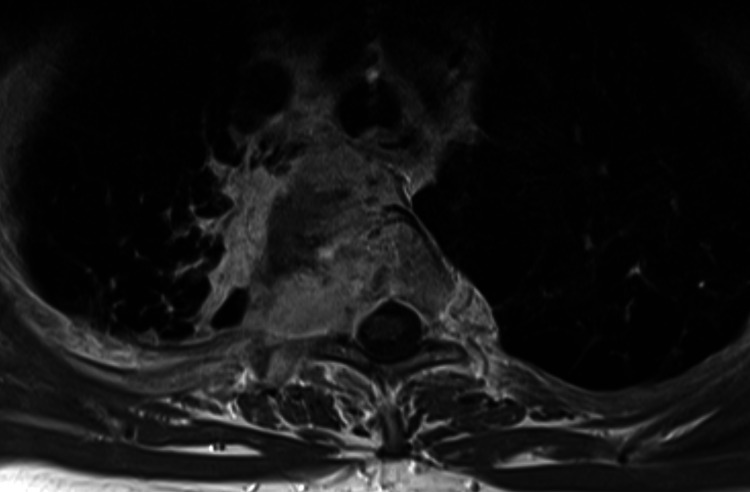
Axial contrast-enhanced T1 TSE MRI at the level of the primary lung tumor. The tumor invades the spine and the spinal canal at the right-hand aspect of the spinal cord. MRI, magnetic resonance imaging; TSE, turbo spin echo

Figures 3, 4 display the supplemental spine MRI.

The final result of the imaging workup was a T4 N2 M1b tumor (right lung, separate 12-mm nodule in the right lower lobe, mediastinal lymph node metastases) with the intraspinal lesion as the only site of distant metastasis (asymptomatic, PET-detected). CT-guided lung biopsy from the main lesion showed poorly differentiated pleomorphic carcinoma with programmed death-ligand 1 (PD-L1) expression in <50% of the tumor cells. Neither EGFR mutations nor ALK translocations were detected. Other molecular tests were not recommended in 2016. No attempt to biopsy other lesions was made. Cerebrospinal fluid biopsy was not obtained either.

**Figure 5:**
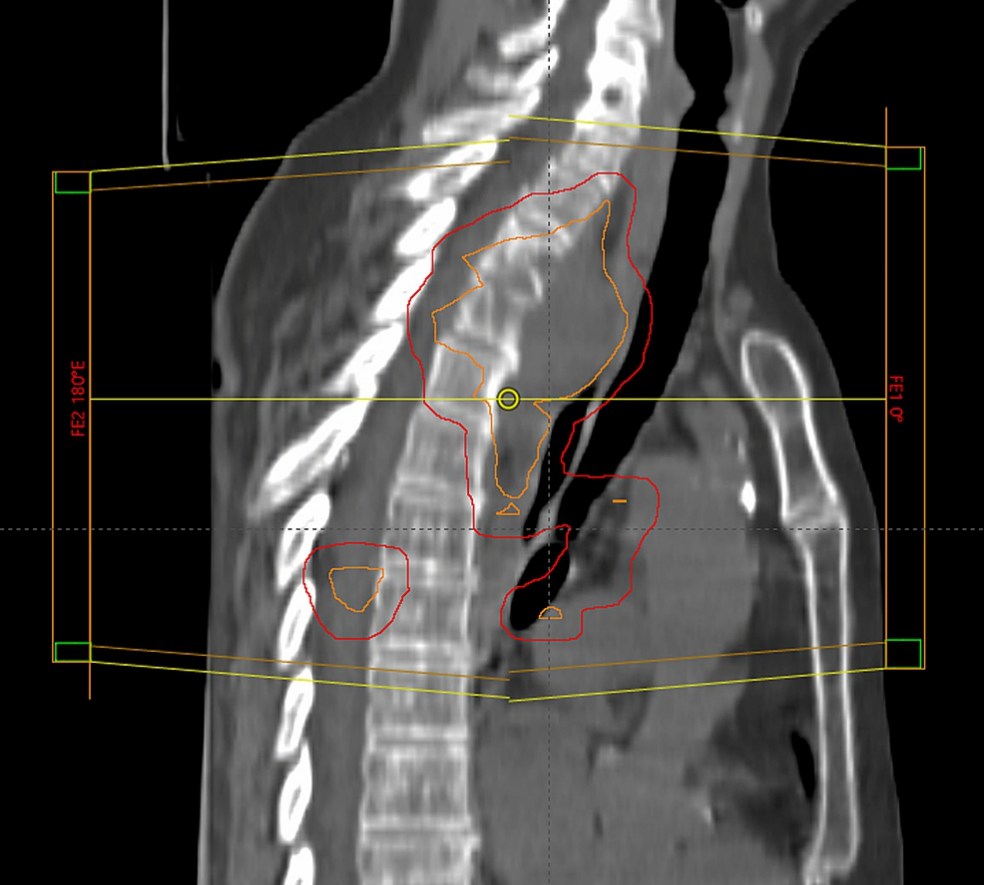
Sagittal treatment field view showing two of four treatment fields. The clinical target volumes are displayed in orange (red contours: planning target volumes).

In accordance with the national guidelines at that time, the multidisciplinary tumor board recommended first-line systemic chemotherapy, and because of pain and the risk of neurological deficits in case of progression, additional palliative radiotherapy was recommended. In September 2016, the patient received her first cycle of carboplatin/vinorelbine. Between the first and second cycles, three-dimensional conformal radiotherapy (30 Gy in 10 fractions of 3 Gy) to all PET-positive lesions was administered (lung, mediastinum, spine; Figure 5).

Due to hematological grade 3 toxicity (anemia, low platelets, neutropenia; Common Terminology Criteria for Adverse Events version 4.0), need for red blood cell transfusion, and additional infection, chemotherapy was stopped after three cycles. CT imaging showed a partial remission with radiation-induced lung reaction, which made it difficult to determine the amount of residual tumor (complete or partial response). No additional PET-CT or MRI scan was ordered. Clinical follow-up and CT imaging continued every three months.

**Figure 6:**
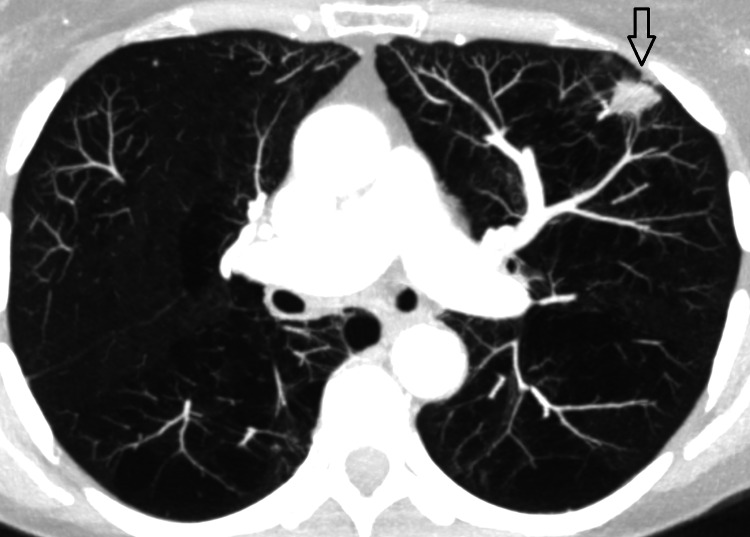
Axial CT scan showing the largest of five lung metastases (anterior part of the left lung, indicated by arrow). CT, computed tomography

Imaging follow-up in June 2017 showed five new metastatic lesions, which were distributed over both lungs (Figure 6).

**Figure 7:**
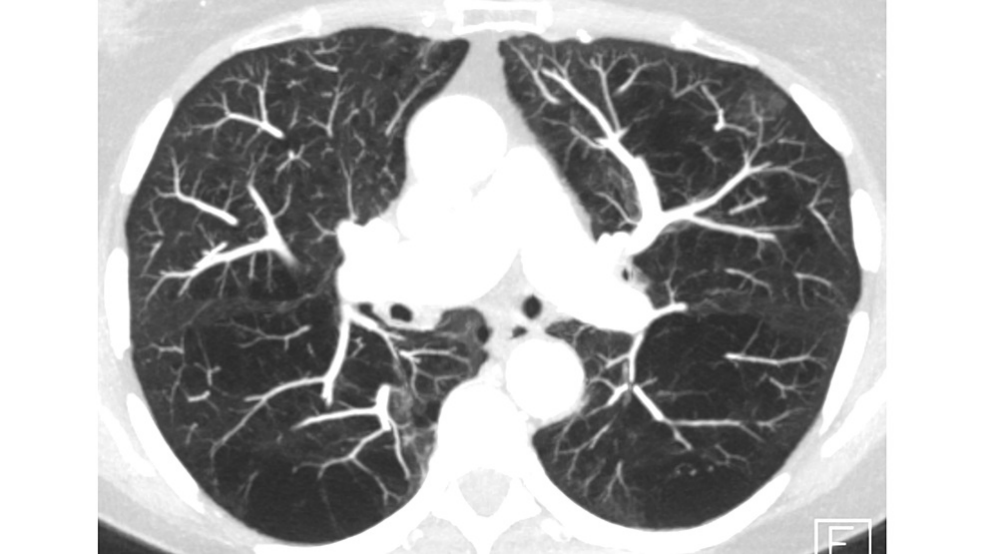
Axial CT scan (September 2021) corresponding to the baseline image before treatment (RECIST criteria: complete remission) CT, computed tomography

The patient was still in good general condition (performance status 1) and started with second-line systemic therapy, Nivolumab (3 mg/kg every other week). A complete response to immunotherapy was recorded, and treatment was tolerated without any serious toxicity. The post-radiation lung changes remained stable and asymptomatic. Due to a change in the national guidelines after routinely scheduled drug price negotiations, Nivolumab was replaced by atezolizumab 1,200 mg every three weeks in June 2018. The clinical course remained unremarkable until June 2019 when the patient successfully completed two years of immunotherapy. Since then, she has remained off-treatment, and during regular follow-up (first at three-month interval, now at six-month interval) neither relapse nor late toxicity has been recorded (Figure 7). The patient is doing well five years after her initial diagnosis of lung cancer.

## Discussion

The present case report describes an uncommon disease presentation (synchronous, limited intraspinal spread) in a common cancer type (locally advanced NSCLC), where distant osseous metastases (including the spine) and brain/leptomeningeal metastases represent scenarios that oncologists are more familiar with. As illustrated in the figures embedded in the case presentation, the primary tumor invaded both the vertebra and spinal canal at the level of the upper thoracic spine. Therefore, it appears possible that the separate, more caudally located ipsilateral lesion detected by PET-CT during the initial staging might be a result of regional rather than hematogenous dissemination (also in the light of absence of classical CNS metastases). Unfortunately, no histological verification was obtained. At the time of diagnosis five years ago, other differential diagnoses had to be considered [12-16]. However, the combination of imaging findings, blood test results, and clinical examination led the multidisciplinary lung tumor board to conclude with metastatic NSCLC (a conclusion that remains valid after five years of follow-up). The treatment intent was defined as palliative and guideline-concordant platinum-doublet chemotherapy was initiated, with planned upfront radiotherapy between the first and second cycles.

Since 2016, treatment algorithms and national guidelines have evolved. It is now also recommended to search for additional targetable tumor cell alterations beyond just EGFR and ALK. As an alternative to the reported management approach, systemic treatment might have been supplemented with upfront radical radiotherapy. Sequential consolidation radiotherapy after induction chemotherapy can also be discussed [17]. Despite the less aggressive and rather cautious approach that actually was chosen, a remarkable response to treatment was observed. Long-term local control after a total dose of only 30 Gy, a classical palliative regimen, is rarely observed [18]. An equally impressive response was induced by second-line immunotherapy, which led to complete disappearance of the lung metastases in this originally PD-L1 positive NSCLC. Of course, the two-year-long phase of second-line treatment might have contributed to the long-lasting local control after irradiation of all PET-positive lesions. Given that the metachronous lung metastases were not biopsied, their molecular profile and level of PD-L1 expression remain unknown. In the phase III OAK study of atezolizumab versus docetaxel, 28% of the patients treated with atezolizumab were alive after at least 24 months [19]. The five-year pooled survival rate was 13% in the nivolumab studies [20]. Nivolumab-treated patients without disease progression at two and three years had an 82% and 93% chance of survival, respectively.

## Conclusions

This case illustrates that outcomes comparable to those observed in patients with limited brain metastases may be observed in patients with localized intraspinal disease and that ICIs play an important role in the management of metastatic NSCLC.
